# The Virtual Screening of the Drug Protein with a Few Crystal Structures Based on the Adaboost-SVM

**DOI:** 10.1155/2016/4809831

**Published:** 2016-04-03

**Authors:** Meng-yu Wang, Peng Li, Pei-li Qiao

**Affiliations:** ^1^School of Computer Science and Technology, Harbin University of Science and Technology, Harbin 150080, China; ^2^School of Software, Harbin University of Science and Technology, Harbin 150080, China

## Abstract

Using the theory of machine learning to assist the virtual screening (VS) has been an effective plan. However, the quality of the training set may reduce because of mixing with the wrong docking poses and it will affect the screening efficiencies. To solve this problem, we present a method using the ensemble learning to improve the support vector machine to process the generated protein-ligand interaction fingerprint (IFP). By combining multiple classifiers, ensemble learning is able to avoid the limitations of the single classifier's performance and obtain better generalization. According to the research of virtual screening experiment with SRC and Cathepsin K as the target, the results show that the ensemble learning method can effectively reduce the error because the sample quality is not high and improve the effect of the whole virtual screening process.

## 1. Introduction

Since the 21st century, the focus of life science has been developed from the experimental analysis and data accumulation to experiments under the guidance of data analysis. Life science is undergoing a transition from analysis of reduction of method to the system integration method [1]. With the completion of human genome project (HGP), more and more three-dimensional structures of important function of biological macromolecules (proteins, nucleic acids, enzymes, etc.) have been parsed [2]. As the amount of data has increased exponentially in recent years, the combination of traditional pharmaceutical field and modern computer technology has become the inevitable result of the development of life science, and virtual screening is the product of this combination. At present, millions of molecules can be screened out by the virtual screening method every day. For each specific target structure, we can get the active compounds in short time. The research object is focused on hundreds of compounds from millions compounds, which can greatly improve the speed and efficiency of the compounds screening and shorten the cycle of new drug research. However, the increasing amount of data makes ordinary computer algorithm unable to maintain a high level, so the machine learning method has gradually entered the view of the scientists due to its reliable and fast performance.

The combination of machine learning and virtual screening has become a hotspot in the field of chemical information and embodies its value in the process of drug discovery, such as searching inhibitors [3], finding novel search chemotypes [4], and predicting protein structures [5]. The number of crystal structures of complex for training is crucial in the method of the combination of virtual screening and machine learning. Relative to the small number of training sets, a larger and more diverse training set can train a more powerful learning mode. However, the crystal structures which can be used for virtual screening always come from X-ray crystal diffraction or the means of NMR [6]. Although the structure is accurate, the high funding and the period limit the speed of resolution, which cannot meet the needs of the virtual screening experiment. So in order to expand the size of the training set, some docking poses of the known active compounds will be added to the training set. Because the docking poses are supposed to include incorrect binding modes, large amounts of negative samples are introduced. The accumulation of the negative samples is possible for producing the imbalanced data set, which is a common phenomenon and of great value in the studies on bioinformatics.

On the prediction of DNA-binding proteins, Song et al. propose an ensemble learning algorithm imDC according to the analysis on unbalanced DNA-binding protein data, which has outperformed classic classification models like SVM under the same situation [7]. Based on the ensemble learning framework, Zou et al. give a new predictor to improve the performance of tRNAscan-SE Annotation, and the experimental results show their algorithm can distinguish functional tRNAs from pseudo-tRNAs [8]. Lin et al. propose merging *K*-means, static selective strategy, and ensemble forward sequential selection on the ensemble learning architecture for hierarchical classification of protein folds with the accuracy reaching 74.21%, which is the state-of-the-art strategy at present [9]. Zou et al. combine the synthetic minority oversampling and *K*-means clustering undersampling to tackle the negative influence brought by imbalanced data sets [10].

Obviously, it is common for bioinformatics studies to face the imbalance data sets. The widely utilized strategies include preprocessing training samples and improving classifiers at present. In this paper, we start from the perspective of improving machine learning algorithm, introducing the ensemble learning method on the basis of simple SVM classifier, using layered combination and iterative weight to enhance the performance of the classifier, so as to reduce the impact of the quality of the sample set. Meanwhile, this paper introduces Random Forest as the experimental baseline to examine the effect of ensemble learning on virtual screening.

## 2. The Quantitative Method

With the rapid development of combinatorial chemistry, bioinformatics, molecular biology, and computer science, the computer aided drug design (CADD) is widely used. Virtual screening as one of the most widely used methods in the CADD, because of its quick and low cost, has been gradually replaced by the high-throughput screening as the main mean of drug screening [11]. In this paper, we will use the virtual screening method to screen the drug protein.

### 2.1. General Process of Virtual Screening

**Figure 1:**
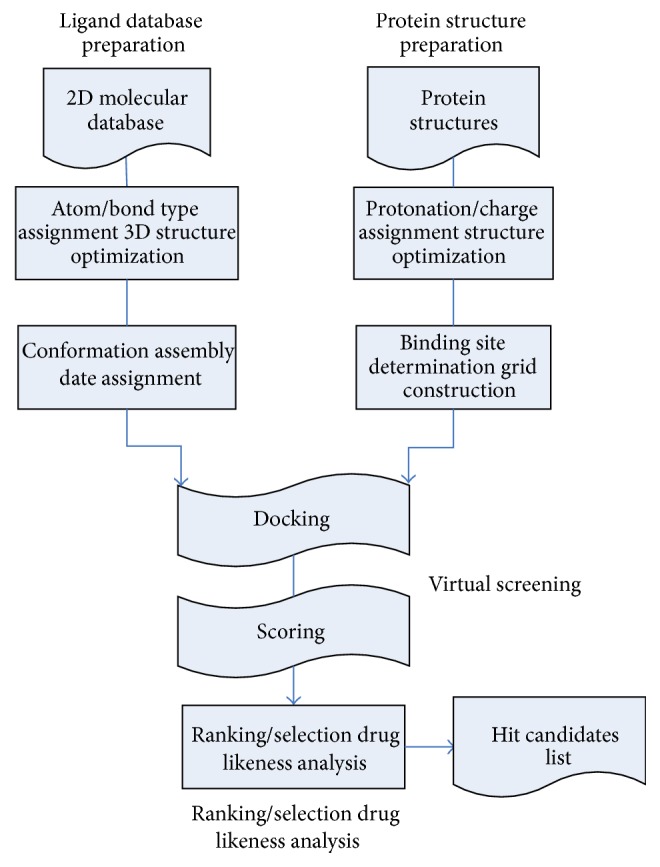
Virtual screening process.

Virtual screening is also known as a computer screen, which is a prescreening of compound molecules on the computer to reduce the number of actual screening compounds and to improve the efficiency of the discovery of lead compounds. The workflow of virtual screening process is shown in Figure 1.

Virtual screening includes four steps: the establishment of the receptor model; the generation of small molecule libraries; the computer screening; the postprocessing of hit compounds.

**Step 1 (the establishment of the receptor model).**

(1) Obtaining macromolecular structure: preparation of protein structure is an important step in the virtual screening. The crystal structures which will be used in the virtual screening can be directly obtained from the PDB or modeling the sequence and structure information of the homologous protein.

(2) Binding site description: the choice of the appropriate ligand binding pocket is very important in molecular docking. There are two ways to choose: (1) we take from the ligand-receptor complex structure directly. (2) If there is no complex structure, we need to manually choose the binding sites according to the experiment information of biological functions such as mutation and combination.

**Step 2 (the generation of small molecule libraries).**

We can use conversion program to translate the two-dimensional structure to three-dimensional structure. The obtained 3D structures can be used for docking process after adding the hydrogen atoms and charges.

**Step 3 (docking and scoring).**

Docking operation is putting every small molecule on ligand binding sites of receptor protein, optimizing the conformation and location of ligand, and making sure of the best combination. To score the best conformation and to rank all compounds according to the scoring, then pick out the small molecules with the highest score from compound library. Docking algorithm aims to predict complex conformation generated by the receptor and the ligand. The purpose of the scoring function is choosing the conformation from candidate set of conformations according to the score. The scoring function will get a lower score if the docking result is more close to the natural compound. However, there is no completely correct scoring function. So far, all kinds of scoring functions used in the existing various docking algorithms are only an approximation to the correct scoring function.

**Step 4 (postprocessing of hit compounds).**

If only use of the sample-scoring model will lead to a huge difference in the final results and sometimes lead to wrong judgment, final results must be analyzed from multiple perspectives and postprocessing. The purpose of this analysis and postprocessing is as accurate as possible to assess protein-ligand binding free energies. The generated complex candidate set is classified, and the error results are distinguished.

As the accuracy of the scoring function in virtual screening has not been properly resolved, in this paper, we use the protein-ligand interaction fingerprint (IFP) to deal with the interactions between target proteins and ligands. The IFP encode the observed interactions between ligand and protein into a binary string of fixed length [12]. The IFP method was originally designed for analyzing ligand docking poses to protein kinases. Based on this method, the atom-based IFP concept was put forward and extended. Each kind of IFP has its own characteristics, whether it is residue-based IFP or atom-based IFP. One-dimensional interaction fingerprint is more likely to be generated and compared with the 3D structure of protein ligand, and it is more suitable for computer aided drug design [13].

### 2.2. The Concept and Calculation Process of Pharm-IF

In this paper, we use a kind of atomic-based fingerprint—Pharm-IF as an aid to verify the theory in this paper. The concept of Pharm-IF is put forward by Sato et al. [14]. The Pharm-IF is calculated from the distances of pairs of ligand pharmacophore features that interact with protein atoms and it can detect important geometrical patterns of ligand pharmacophore.

**Figure 2:**
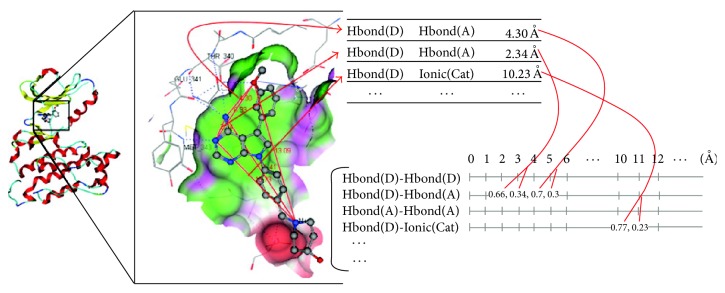
The calculation process of Pharm-IF [14].

The calculation of Pharm-IF can be divided into the following three steps as Figure 2 shows.

**Step 1 .**

To detect the protein-ligand interactions from complex structures, interactions can be classified into six types: (1) hydrogen bond with ligand acceptor; (2) hydrogen bond with ligand donor; (3) hydrogen bond in which the roles of ligand and protein atoms could not be determined; (4) ionic interaction with ligand cation; (5) ionic interaction with ligand anion; (6) hydrophobic interaction.

**Step 2 .**

To create all possible interaction pairs, each interaction pair is characterized by the pharmacophore features of the ligand atoms and their distance. To calculate the resulting matrix, each interaction pair is assigned to the corresponding bin. In an interaction pair of two hydrogen bonds, ligand atoms will be assigned to the vector corresponding to this hydrogen bond pair if ligand atoms are a donor and an acceptor that are 4.3 Å apart from each other. For example, in order to describe the distance of 4.3 Å in the interaction, 0.7 is assigned to the bin of 4 Å and 0.3 is assigned to the bin of 5 Å.

**Step 3 .**

The result matrix is calculated by the summation of the values of all of the interaction pairs. The formula of Pharm-IF calculation is as follows:

(1) Ht,k=∑i∈ItAki,

(2) Aki=0,if  k−di≥1,1−k−di,otherwise.

In formula (1), *H* stands for the interaction fingerprint of a protein-ligand complex by Pharm-IF. *t* is the pair of six types of pharmacophore features. *k* = 1,2, 3,… stands for the corresponding bins of the distances (Å) between ligand atoms. *I*_*t*_ represents the fact that the whole set of the interactions are classified as type *t*, and *i* represents an element in *I*_*t*_. In formula (2), *d*_*i*_ represents the distances between ligand atoms of *i* (Å).

### 2.3. Cathepsin K and SRC

This paper selects Cathepsin K and SRC as the target for screening. These two kinds of proteins are the hotspot in the field of pharmaceutical drug targets and both of them do not have enough experimentally determined protein-ligand complex structures for virtual screening. Therefore, it is necessary to add some docking poses in the training set and these docking poses will influence the virtual screening efficiency.

Protooncogene tyrosine-protein kinase SRC, also known as protooncogene c-Src or simply c-Src, is a nonreceptor tyrosine kinase protein that in humans is encoded by the SRC gene. The SRC family kinase is made up of 9 members: LYN, FYN, LCK, HCK, FGR, BLK, YRK, YES, and c-SRC. The SRC widely exists in tissue cells and it plays an important role in the process of cell metabolism, regulation of cell growth, development, and differentiation process by interacting with the important molecules in the signal transduction pathways. The c-Src is made up of 6 functional regions: SRC homology 4 (SH4) domain (SH4 domain), unique region, SH3 domain, SH2 domain, catalytic domain, and short regulatory tail. When SRC is inactive, that will cause intermolecular interactions between the phosphorylation TYR527 (tyrosine group 527) and SH2 domain. At the same time, the SH3 domain will combine with the proline-rich SH2 kinase link domain. When Tyr527 is dephosphorylated and Tyr416 is phosphorated, links between these molecules will break, and the SRC protein is activated. The SRC causes a series of biological effects by participating in many signal transduction pathways through a variety of receptors and this kind of protein is closely associated with a variety of cancers. The activation of the c-Src pathway has been observed in about 50% of tumors from colon, liver, lung, breast, and the pancreas. As a drug target, a number of tyrosine kinase inhibitors treating c-Src tyrosine kinase (as well as related tyrosine kinases) as target have been developed and put into use [15].

Cathepsin K is a lysosomal cysteine protease belonging to the papain superfamily and it has been cloned in 1999. The gene location is lq21.2, the length of the transcript is 1.7 kb, and it consisted of 8 extrons and 7 introns. The protein expression is in the osteoclasts and included in the bone resorption. In the process of bone resorption, the acid will dissolve the hydroxyapatite and the organic ingredients in the bone matrix will be separated and degraded by Cathepsin K. Cathepsin K has strong activity of collagenase in acid environment and it has been found that it plays a role in a variety of pathological phenomena at present such as rheumatoid arthritis, tumor invasion and metastasis, inflammation, and osteoporosis. The function of Cathepsin K in osteoclast has been recognized; therefore, many labs treat their inhibitors as a drug target for the treatment of osteoporosis. At present, the first choice of antiabsorption treatment is bisphosphonates, which can reduce the risk of nonvertebral and vertebral fractures. However, the long-term using of bisphosphonates may produce adverse reactions: esophageal stimulus symptoms, hypocalcaemia, kidney irritation, and so on. In addition, bisphosphonates not only prevent bone loss but also inhibit bone formation at the same time, so the new replacement therapy drugs are more meaningful [16].

## 3. Classification Algorithm Based on the Adaboost-SVM

Currently, SVM can deal with many problems, such as small size of samples, nonlinearity, or high dimensions. Based on the statistical learning theory, it has a simple mathematical form, fast training method, and good generalization performance. It has been widely used in data mining problems such as pattern recognition, function estimation, and time series prediction. Under the condition that the quality of sample set is not very low, even if we do not make any improvements, we can get a good result. The learning mechanism of SVM provides a lot of space to improve the classification model. In addition, one major advantage of SVM is using of convex quadratic programming, which provides only global minima and hence avoids being trapped in local minima, so in this paper we use SVM as the base classifier. There have been a large number of literatures about the SVM; this paper only gives a simple introduction. The basic process of SVM classification problems is as follows.

For a given sample set *L* = {(*x*_1_, *y*_1_), (*x*_2_, *y*_2_),…, (*x*_*n*_, *y*_*n*_)} and *x*_*i*_ ∈ *R* ^*d*^ *y*_*i*_ ∈ {1, −1}, *i* = 1,2,…, *n*, *y*_*i*_ stands for the categories of sample *x*_*i*_, *d* is the sample number, and *n* is the training sample number. If the input vector set is linearly separable, then the input vector set can be separated by a hyperplane. The hyperplane can be expressed as

(3) $$\begin{array}{c} w\cdot x-b=0. \end{array}$$

*w* is the normal vector of the hyperplane and *b* is offset. The SVM learning problem is minimizing the objective function:

(4) $$\begin{array}{c} \min\phi \left(\;w\;\right)=\frac{1}{2}{\left\|\;w\;\right\|}^{2}+C\left(\;\overset{n}{\underset{i=1}{\sum }}\xi_{i}\;\right). \end{array}$$

This meets the condition

(5) $$\begin{array}{c} y_{i}\left(\;wx_{i}+b\;\right)\geq 1-\xi_{i},\;i=1,2,\ldots ,n. \end{array}$$

Here, (1/2)‖*w*‖^2^ is structure complexity, *C*(∑_*i*=1_^*n*^ *ξ*_*i*_) stands for empirical risk, and *ξ*_*i*_ presents the slack variable. *H* is a constant which is punishment factor of samples wrongly classified. For the situation of linear inseparable the main idea of SVM is used to map the feature vector to the high dimensional feature space and constructs an optimal hyperplane in the feature space.

To get the change of *ϕ*, *x* in space of *R* ^*n*^ mapped into *H*:

(6) $$\begin{array}{c} x⟶\phi \left(\;x\;\right)={\left(\;\phi_{1}\left(\;x\;\right),\phi_{2}\left(\;x\;\right),\ldots ,\phi_{i}\left(\;x\;\right)\;\right)}^{Τ}. \end{array}$$

Eventually we can decide optimization classification function:

(7) fxsgn⁡w·ϕx+b=sgn⁡∑i=1naiyiϕxi·ϕx+b.

In our work, Radial Basis Function (RBF) is taken as the kernel function of SVM, and the mathematical description of this kernel is given below:

(8) $$\begin{array}{c} K\left(\;x_{i},x_{j}\;\right)=e^{-{\left\|x_{i}-x_{j}\right\|}^{2}/2\sigma^{2}}. \end{array}$$

Relative to other classifiers, the SVM is more stable and less affected by the quality of sample set. However, the sample categories imbalance of data set and the high complexity of the data set will destabilize the classifier and the instability of the classifier will directly affect the final classification result. In this paper, we introduce the Adaboost mechanism in ensemble learning to divide one classification process into several layers of weak classifier based on SVM.

The key of the combination of Adaboost and SVM is to find a suitable Gauss width *σ* value for each component. If the *σ* value is relatively large, the component classifier is too weak, and the final classification performance is decreased. On the other hand, if the *σ* value is relatively small, which makes the component classifier robust, and the error of component classifier is highly correlated, the difference is small, so that the ensemble learning is invalid. Even more importantly, *σ* value is too small which will lead to overfitting and resulting in a greatly reduced generalization. Therefore, in this paper, the standard deviation of the sample set of each component classifier is used as the *σ* value of the component classifier to control the classification accuracy of the component classifier; thus SVM based Adaboost classifier is obtained. The program used in this paper is not an open source, so we need to explain some key parameters. We list the values of *σ*, *C* and other parameters in Table 1.

The specific process of the algorithm is as follows.

(1) RBFSVM presents the SVM with the RBF kernel; *T* presents the number of iterations required in the Adaboost process.
(2) Initialization: initialize the weights of each sample: *w*_1_(*i*) = 1/*n*, *i* = 1,2,…, *n*.
(3) For *t* = 1,2,…, *T*:
 for each *h*(*x*_*i*_), calculate the weighted error:
  (9) $$\begin{array}{c} {\varepsilon_{t}}^{\left(i\right)}=\overset{n}{\underset{j=1}{\sum }}D_{i}\left|\;h\left(\;X_{j}\;\right)-y\left(\;j\;\right)\;\right|. \end{array}$$
 Choose a feature with the lowest weighted error rate *ε*_*j*_ and save its corresponding SVM model. Calculate the selected weak classifier's weight:
  (10) $$\begin{array}{c} a_{t}=\frac{1}{2}\ln\left(\;\frac{1-\varepsilon_{t}}{\varepsilon_{t}}\;\right). \end{array}$$
 Update sample weights according to *a*_*t*_:
  (11) Dt+1iDtiZtFEt=DtiZte−αtif  htxi=yi,eαtif  htxi≠yi.
 And the normalized parameters are
  (12) $$\begin{array}{c} \overset{n}{\underset{i=1}{\sum }}D_{t}\left(\;i\;\right)=1. \end{array}$$
(4) Use strong classifier *H* integrated by SVM weak classifier to training set:
  (13) $$\begin{array}{c} H\left(\;x\;\right)=\mathrm{sign}\left[\;\overset{n}{\underset{i=1}{\sum }}a_{t}h_{t}\left(\;x\;\right)\;\right]. \end{array}$$

For the Adaboost-SVM, we set the parameters in Table 2, and the basic SVM parameters are the same as Table 1.

Thus compared to the single machine learning algorithm, ensemble learning method requires that each base classifier should be independent from the others. The probability of sample misclassification should be less than 0.5. In the ensemble learning method, all classifiers will work together to solve one problem and this can also reduce the impact of the quality of sample set to the virtual screening effect.

## 4. Experiment and Analysis

In order to verify the validity of the proposed method in this paper, besides the crystal structure of PDB, we also combine the data from the PubChem database and the StARLITe database and the enrichment factor (EF) and the ROC curve are used to evaluate the effect of virtual screening and the machine learning to ensure the effectiveness of the method. PubChem is a database of chemical molecules and their activities against biological assays. StARLITe is a database containing biological activity and/or binding affinity data between various compounds and proteins and it is one of the databases that can be directly used in data mining.

All the crystal structures used in this experiment are from PDB database; the material is available free of charge via the Internet at http://www.rcsb.org/. All the training set decoys are from PubChem data set; the material is available free of charge via the Internet at http://pubchem.ncbi.nlm.nih.gov/. We thank laboratory colleagues for providing the StARLITe data.

### 4.1. Data Set

In order to cooperate with machine learning, in this paper, we construct a set of training sets and test sets of these two target proteins for machine learning, which include the decoys and known active compounds. In the training set, we selected the experimentally determined complex structures of these two kinds of proteins from the PDB as the positive samples. The Protein Data Bank (PDB) is a crystallographic database for the three-dimensional structural data of large biological molecules. The data in the PDB is submitted by biologists and biochemists around the world by the experimental means such as X-ray crystallography, NMR spectroscopy, or, increasingly, cryoelectron microscopy. To expand the training set, we randomly and respectively selected 1, 3, 5, 10, 20, 40, 60, and 80 active compounds from the known active compounds for which crystal structures with their targets were not determined and this process is repeated 10 times. For each of the selected compounds, we used the GLIDE to generate five docking poses and mixed them into the training set. Among them, the crystal structures of these two proteins used in docking experiments are selected from the PDB with protein-inhibitor compounds with high inhibitor activity and high resolution crystal structure. The entry 2h8h, SRC kinase in complex with a quinazoline inhibitor, the resolution 2.30 Å, was selected for SRC. The entry 1u9w, crystal structure of the cysteine protease human Cathepsin K in complex with the covalent inhibitor NVP-ABI491, the resolution 2.20 Å, was selected for Cathepsin K. We used the SP mode of GLIDE to generate the docking poses of the decoys and the active compounds for which crystal structures were not experimentally determined. For the preparation of the docking, we use the Protein Preparation Wizard to add the hydrogen atoms of the protein and optimize their positions. Using Pipeline Pilot of SciTegic to enumerate the tautomer, stereoisomers and protonation/deprotonation form at pH 7.4 of the active and decoy compounds. Then, the additional ring conformations of the compounds were generated by LigPrep. Then the GLIDE score was used to select five poses for each compound in the docking results. In this paper, five docking poses of each active compound as the positive examples were chosen by the GLIDE score because this procedure would generate higher enrichment factors in a preliminary test than using one pose of each active compound. Other settings of GLIDE were set to the default values. In this paper, we use the averages of the 10% EF and the ROC score of the 10 trials to evaluate the screening efficiencies of the learning models using each number of docking poses. Then, select the docking poses of 2000 decoy compounds from them as the negative samples, randomly. Each decoy compound was docked to the target proteins by the same way as mentioned above.

After the completion of the training set, we set out to build the test set. First, choose the active compounds of the target proteins (IC_50_ ≤ 10 *μ*m) from StARLITe and divide them into 100 clusters. Dividing strategy is that hierarchical clustering with Ward method based on the Euclidean distance between their 2D structure fingerprints. The compound with the highest inhibitory activity was selected from each cluster. Dock the 100 active compounds obtained for each target to their target protein and five docking poses for each active compound were used as positive samples of the test set. The docking way and target protein crystal structures are same as those mentioned above. Then, use selection strategy of negative sample of the training set to choose the decoys for the test set.

After the completion of the date preparation, we will use the Pharm-IF to quantify the training set. Then, treat the data as the input of machine learning algorithms to get the corresponding learning model. The learning model obtained will be used to test set, respectively.

From Table 3, it can be seen that the proportion of negative samples and positive samples is up to 20 : 1, which leads to significant imbalanced-data problem, and the motivation of our work is to address this issue.

### 4.2. The Evaluation Index of the Machine Learning Combined with Virtual Screening

At present, the virtual screening and machine learning have their own evaluation index [17]. The enrichment factor (EF) is one of the most famous measures for evaluating the screening efficiency. EF is usually used to evaluate the early recognition properties of screening method and it can indicate the ratio of the number of obtained active compounds by in silico screening against that generated by random selection at the predefined sampling percentage. The calculation method of EF is as follows:

(14) $$\begin{array}{c} \text{EF}=\frac{{\text{Hits}}_{s}/N_{s}}{{\text{Hit}}_{t}/N_{t}}. \end{array}$$

**Figure 3:**
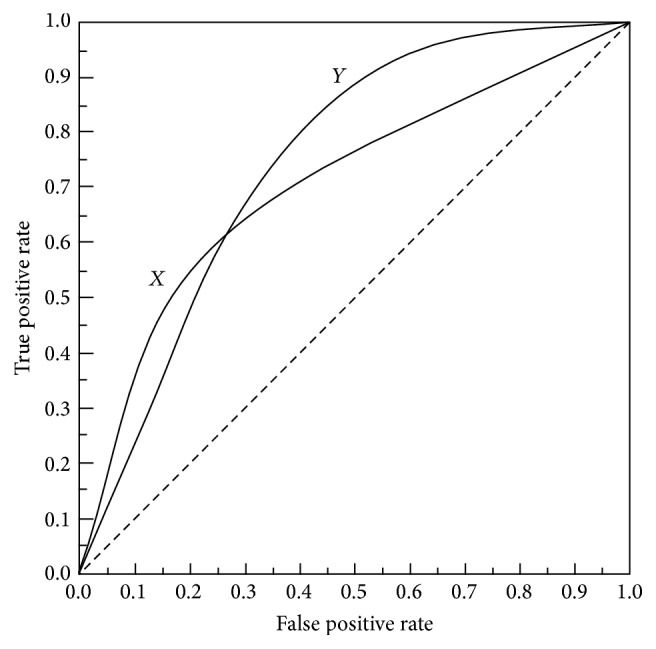
Two ROC curves *X* and *Y*.

Here, Hits_*s*_ represents the number of active compounds in the sample, Hit_*t*_ is the number of active compounds tested, *N*_*s*_ is the number of compounds sampled, and *N*_*t*_ is the number of all compounds. In the actual drug discovery, only a small part of the compound is filtered by computer. In general, 0.01–1% of the compounds will be selected from a huge compound database (10000–1000000 compounds) in the actual virtual screening process. Since the number of test sets in this experiment is far from reaching this order of magnitude, we use 10% EF to carry out this test in order to reduce the deviation of the early evaluation. EF has only specific sampling proportion screening efficiency; therefore this paper also introduced the ROC curve and AUC value to assess the entire range of sampling (0–100%). The ROC curve (receiver operating characteristic curve) is a graphical method to show the tradeoff between false positive rate and true positive rate of classifier. As shown in Figure 3, in ROC curve, the true positive rate (TPR) is plotted along the *y*-axis, while the false positive rate (FPR) is displayed on the *x*-axis. Although the ROC curve can directly reflect the effect of the machine learning model, we also need a kind of numerical method, the AUC (area under ROC curve) value, to evaluate the effect of the model in the practical application. The AUC value indicates the area under the ROC curve and it is more intuitive and accurate. The calculation method of AUC value is as follows:

(15) $$\begin{array}{c} \text{AUC}=\overset{1}{\underset{0}{\int }}\frac{\text{TP}}{P}d\frac{\text{FP}}{N}=\frac{1}{P\cdot N}\overset{N}{\underset{0}{\int }}\text{TP}\;d\;\text{FP}. \end{array}$$

Among all the variables of formula (15), *P* stands for the positive samples, *N* represents the negative samples, TP (true positive) stands for the active compounds that are classified correctly, and FP (false positive) stands for the misclassification of active compounds.

AUC values are between 0.5 and 1.0; if the model is perfect, the AUC value is 1; if the model is only a random guess, the AUC value is 0.5. If a model is better than another, AUC value of the better one is higher. ROC curves and AUC are not affected by imbalance distribution of data class and normal distribution of the data. In addition, the AUC value allows a middle state and experimental results can be divided into multiple ordered classification.

### 4.3. The Analysis of Experimental Results

According to the experiment, we used three different classification methods (SVM, Adaboost-SVM, and RF) to compare the two kinds of target proteins for virtual screening experiment. As the baseline algorithm, RF is also developed based on the machine learning libs of the authors' lab, and the parameters of RF are set in Table 4.

**Figure 4:**
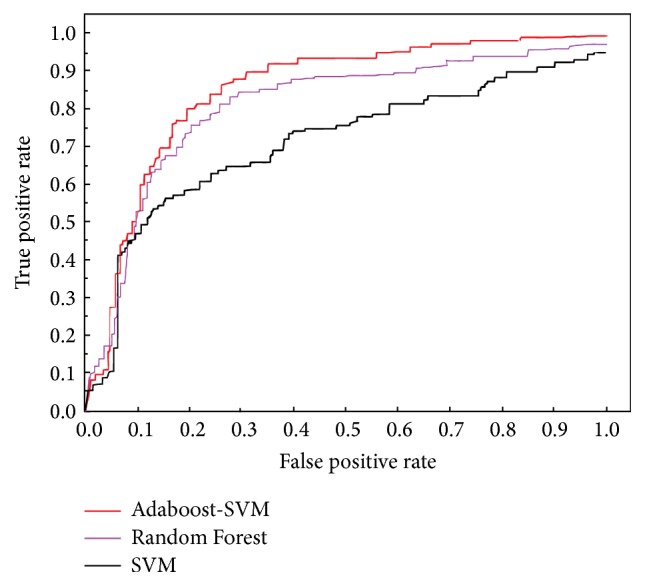
Comparative experiment of SRC.

**Figure 5:**
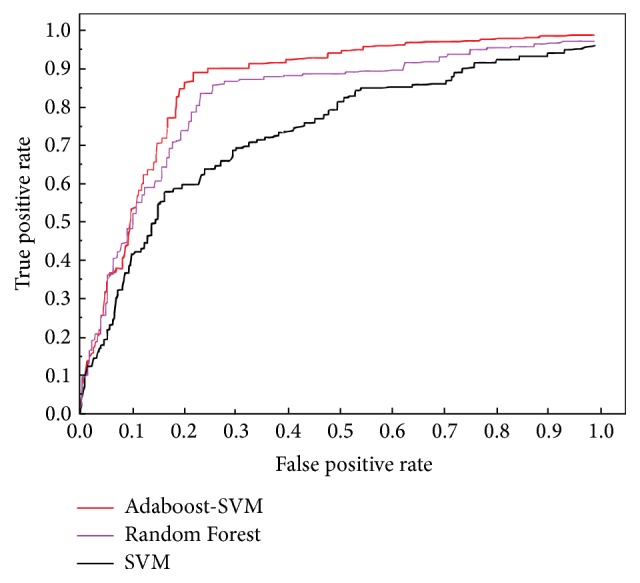
Comparative experiment of Cathepsin K.

The ROC curves from the comparative experiments on these two kinds of data sets using ensemble learning (compared with SVM) are as those in Figures 4 and 5.

Table 5 shows the 10% EF of the two methods and calculates the AUC value.

Aiming at the problem of sample set quality, the Adaboost method gives the same weight value to each training data; the sample weight represents the probability of the data treated as the training set by a weak classifier. At each iteration, the Adaboost algorithm will modify the weight value of the sample; if the training sample is correctly classified in this iteration, the weight value of the sample will be reduced; that is, the probability of being treated as the training sample is reduced in the next iteration. On the contrary, if the training sample is misclassified in the current base classifier, the weight value of the sample will be increased, and the probability of being treated as the training sample will be increased in the next iteration. In this way, the weak classifier will pay more attention to the serious misclassification of training set. The experimental results above show that Adaboost-SVM has notably outperformed RF. This observation indicates that on both 10% EF and AUC ensemble learning based virtual screening has shown its ability of noise resistance, under the situation that the amount of structure samples is limited; thus the better screening results are obtained.

## 5. Conclusion

In this paper, we use ensemble learning method to solve the problem caused by the quality of the training set. This method mainly uses Pharm-IF to encode protein-ligand interactions as a binary form and then uses the improved SVM algorithm, Adaboost-SVM, and Random Forest to classify the data. The idea of ensemble learning in dealing with data classification problem is to get a number of weak classifiers which are independent of each other and then use an effective method to combine these independent weak classifiers. By comparing the experimental results, after the Adaboost-SVM is used as the classifier, 10% EF for the SRC model increased from 4.7 to 5.5, and the AUC value increased from 0.734 to 0.821, 10% EF of Cathepsin K model increased from 3.9 to 4.8, and the AUC value increased from 0.683 to 0.802. It can be observed from the results that, comparing with the naïve SVM, Random Forest has obtained better performance on both 10% EF and AUC: 10% EF is improved to 5.3 and 4.5 on SRC and Cathepsin K, respectively, and AUC is improved to 0.805 and 0.783, respectively. As a classic ensemble learning algorithm, Random Forest has shown that ensemble learning is able to get better results on the imbalanced data set with satisfying robustness. Nevertheless, the performance of Random Forest is lower than Adaboost-SVM, and we will continue investigating the reasons in our future work. Compared with the traditional method, the proposed method is more significant for the improvement of the accuracy of the virtual screening model. In the future work, the problem of improving the accuracy of virtual screening should be further studied from two aspects: virtual screening theory and computer theory. Although the status of virtual screening is gradually increasing, the problem of virtual screening false positive rate is still to be solved. The speed of laboratory determination of protein structure has been unable to catch up with the needs of drug development, therefore, in the virtual screening it will often encounter problems similar to this paper, so there are still many improvements in the algorithm. For example, the selection of kernel function will directly affect the performance of the classifier. In view of the problem of this kind of data set, we should set up a special kernel function to adapt to the characteristics of the data set.

## Figures and Tables

**Table 1:** SVM parameter setting.

Parameter name	Parameter values
SVM type	C-SVM
Class number	2
Kernel function	RBF
The degree in kernel function	3
*σ* in kernel function	0.001
Coast factor	5
Cache size	500 MB
Tolerance in the termination criteria	0.001
The weight value of penalty factor for all kinds of samples	1
Cross validation	5

**Table 2:** Adaboost-SVM parameter setting.

Parameter name	Parameter values
Ensemble learning type	Adaboost
Class number	2
The basic classifier type	C-SVM
Number of classifiers per layer	100
The max false alarm rate	0.5
The min hit rate	0.9
Number of iterations	5
Weight trim rate	0.9
Cache size	500 MB

**Table 3:** Experimental data structure.

Date set	Positive sample	Negative sample	Total
Training set	100	2000	2100
Test set	100*∗*5 = 500	2000*∗*5 = 10000	10500

**Table 4:** Random Forest parameter setting.

Parameter name	Parameter values
Tree number	1000
Node size	5
The number of different descriptors tried at each split	50

**Table 5:** Experimental comparison of SVM, Adaboost-SVM, and Random Forest.

Algorithm	Target protein	10% EF	AUC
SVM	SRC	4.7	0.734
	Cathepsin K	3.9	0.683

Adaboost-SVM	SRC	5.5	0.821
	Cathepsin K	4.8	0.802

Random Forest	SRC	5.3	0.805
	Cathepsin K	4.5	0.783
